# A Systematic Review of Decision Aids in Hematologic Malignancies: What Are Currently Available and What Are We Missing?

**DOI:** 10.1093/oncolo/oyac231

**Published:** 2022-11-07

**Authors:** Janice Zhao, Maya Abdallah, Chandrika Sanapala, Erin Watson, Marissa LoCastro, Daniel A Castillo, Daniel Richardson, Thomas W LeBlanc, Kah Poh Loh

**Affiliations:** Division of Hematology/Oncology, Department of Medicine, James P. Wilmot Cancer Institute, University of Rochester Medical Center, Rochester, NY, USA; Section of Hematology and Medical Oncology, Boston University School of Medicine, Boston, MA, USA; Section of Hematology and Medical Oncology, Boston University School of Medicine, Boston, MA, USA; Department of Psychology, Princeton University, Princeton, NJ, USA; School of Medicine and Dentistry, University of Rochester, Rochester, NY, USA; Edward G. Miner Library, University of Rochester School of Medicine and Dentistry, Rochester, NY, USA; Division of Hematology, Department of Medicine, Lineberger Comprehensive Cancer Center, University of North Carolina, Chapel Hill, NC, USA; Department of Medicine, Hematologic Malignancies and Cellular Therapy, Duke University School of Medicine Durham, Durham, NC, USA; Division of Hematology/Oncology, Department of Medicine, James P. Wilmot Cancer Institute, University of Rochester Medical Center, Rochester, NY, USA

**Keywords:** decision aids, hematologic malignancies, shared decision-making

## Abstract

**Background:**

Patient decision aids (PDAs) are tools designed to facilitate decision-making. In this systematic review, we summarized existing studies on the development and evaluation of PDAs for patients with hematologic malignancies.

**Patients and Methods:**

We followed the Preferred Reporting Items for Systematic Reviews and Meta-Analyses (PRISMA) guidelines. We searched for articles in PubMed, Embase, Web of Science, Cochrane Central Register of Controlled Trials, and ClinicalTrials.gov. We included studies, abstracts, and clinical trial protocols available in English involving PDAs for patients age ≥18 diagnosed with a hematologic malignancy and/or their caregivers. Data were summarized using descriptive statistics.

**Results:**

Of the 5281 titles/abstracts screened, 15 were included: 1 protocol, 7 abstracts, and 7 full-texts. Six were PDA developmental studies, 6 were pilot studies, and 3 were randomized trials. PDA formats included electronic with web content, videos, and/or audio, questionnaires, bedside instruments, and a combination of various formats. Average participant age ranged from 36.0 to 62.4 years. Patients and caregivers identified efficacy, adverse effects, cost, and quality of life as important decision-making factors. PDAs were associated with increased knowledge and patient satisfaction as well as decreased decisional conflict and attitudinal barriers. Research on PDAs for adult patients with hematologic malignancies and their caregivers is limited. Among the studies, PDAs appear to support patients in shared decision-making.

**Conclusion:**

While current literature examining the use of PDAs for adults with hematologic malignancies is limited, the positive impact of PDAs on shared decision-making and patient outcomes warrants additional research in this field.

## Teaser

Patient decision aids (PDAs) are tools designed to facilitate decision-making. This review summarizes existing studies on the development and evaluation of PDAs for patients with hematologic malignancies.

**Implications for Practice**

There are limited high–quality studies that assess the use of patient decision aids for adults with hematologic malignancies. While limited, existing studies appear to show that PDAs improve outcomes such as knowledge, satisfaction, and decisional conflict.

## Introduction

Hematologic malignances include a large number of various conditions such as leukemia and lymphoma. In 2016, there were 467 000 new cases of leukemia and 461 000 new cases of non-Hodgkin lymphoma worldwide. Between 2006 and 2016, the number of incident cases of leukemia and non-Hodgkin lymphoma increased by 26% and 45%, respectively^1^. There are many decisions that patients diagnosed with hematologic malignancies face, one of which being treatment choice. Treatment options include chemotherapy, immunotherapy, radiation therapy, stem cell transplant, and active surveillance. In addition, patients may face decisions regarding clinical trial participation.

Shared decision-making is a process in which patients and oncologists make decisions together after considering the options, their associated benefits and arms, and patient’s values^2^. Studies have shown that shared decision-making regarding cancer treatment is associated with greater patient knowledge of treatment options as well as greater patient satisfaction with their care experience as a whole^3,4^. A study examining shared decision-making in cancer care found that patients who felt like they engaged in shared as opposed to physician-controlled decision making were more likely to rate their quality of care as “excellent.” These patients also rated physician communication more favorably^3^.

Patient decision aids (PDAs) are tools designed to help guide and support patients in the shared decision-making process. These aids, which can be found in a variety of formats such as pamphlets, interactive websites, and videos, are developed to help patients and their caregivers make well-informed and personalized decisions consistent with their values and goals^5,6^. These decisions can include choosing types of cancer treatment [eg, intensive versus lower intensity treatment in acute myeloid leukemia (AML), whether to enroll on clinical trial, or supportive care services or resources such as palliative care]. A systematic review examining 105 studies on decision aids found that PDAs improved participants’ knowledge and improved congruency between informed values and care choices^7^. In addition, PDAs reduced decisional conflict due to feeling uninformed and reduced the number of participants who were passive in decision making^7^. Due to the variability that exists among PDAs, the International Patient Decision Aid Standards (IPDAS) Collaboration was established in 2003 to improve both the effectiveness and the quality of PDAs. The IPDAS Collaboration has developed criteria that can be used by both developers and users of PDAs to evaluate the quality of a PDA^8^.

There have been systematic reviews examining the role of PDAs in cancer treatment decision making for various malignancies including breast cancer, colon cancer, and prostate cancer^9-11^. While there are numerous decision aids designed for patients with hematologic malignancies, to the authors’ knowledge, there is not a comprehensive systematic review examining the available decision aids for patients with hematologic malignancies. Compared to solid tumors, there is a greater degree of uncertainty in hematologic malignancies^12^. In addition, many treatments options have become available in the last decade for patients with hematologic malignancies^13,14^. Navigating these options is increasingly complicated for patients and oncologists^15^. In some circumstances, decisions are preference-sensitive (eg, upfront chemotherapy for an older adult with acute myeloid leukemia)^16,17^. A better understanding of the landscape of decision aids developed for this population will help identify gaps. The objective of this study was to conduct a systematic review and synthesize studies on decision aids designed for patients age 18 or older with a hematologic malignancy and/or their caregivers. This review examined available decision aids for hematologic malignancies as well as the effects of these tools and identified gaps in the current literature available on this topic.

## Subjects and Methods

### Data Sources and Search

We completed this systematic review following the Preferred Reporting Items for Systematic Reviews and Meta-Analyses (PRISMA) guidelines^18^. We registered our study protocol with PROSPERO, an international database of registered systematic reviews (CRD42020203423). Using a search strategy developed with the assistance of a medical librarian, we searched for articles discussing decision aids and hematologic malignancies in 5 databases, which included PubMed, Embase, Web of Science, Cochrane Central Register of Controlled Trials, and ClinicalTrials.gov. We included terms for “decision making,” decision support,” “hematologic neoplasms,” “plasma cell neoplasms,” “leukemia,” “lymphoma,” “patient,” and “caregivers” and used the exclusion filters recommended by McGill to remove studies indexed as adult only on PubMed and Embase^19^. The search included any articles published between inception and August 26, 2020 (Supplementary Table S1).

### Eligibility Criteria

We included studies based on the following criteria: (1) Development or evaluation of a decision aid; (2) Decision aid was designed for patients age 18 or older diagnosed with a hematologic malignancy and/or their caregivers; (3) Study design was a randomized controlled trial, pilot study, or qualitative study; and (4) Abstract, full text, or clinical trial protocol (e.g., listed on clinicaltrials.gov) was available. We included abstracts and clinical trial protocols due to limited published studies. Eligible studies included educational interventions that increased readiness for shared decision-making. Exclusion criteria were: (1) Decision aid that was designed solely for patients with non-hematologic malignancies; (2) Decision aid was designed primarily for patients less than the age of 18; and (3) Non-English studies. Observational studies and case studies were excluded from this review. These studies were excluded because they do not evaluate the feasibility, acceptability, or efficacy of the decision aids. For all abstracts and clinical trial protocols identified from our search strategy, we searched for full texts if they were not already included to ensure that studies were not missed in our search strategy.

### Study Selection

**Figure 1.**
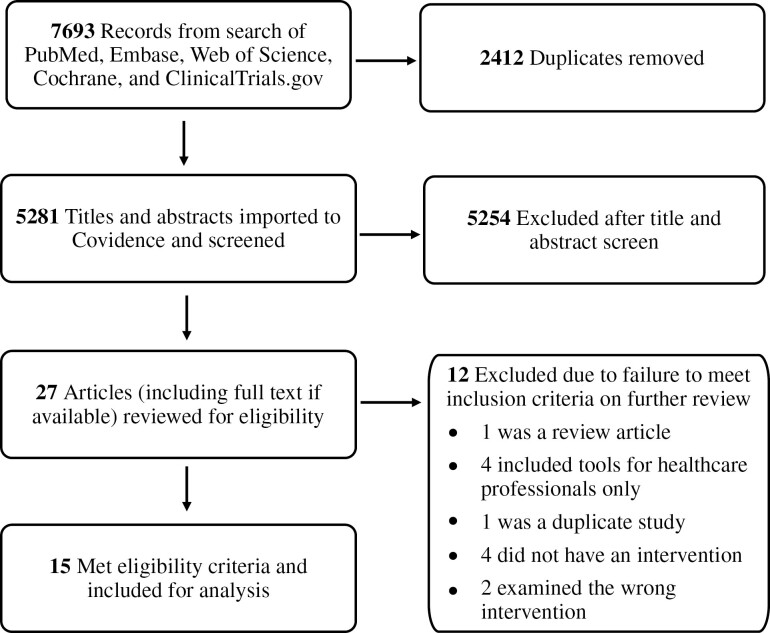
Study selection flowchart.

After using our search strategy to identify potential studies, we exported these studies into Endnote to allow for duplicate removal. We then imported study titles into Covidence (Veritas Health Innovation, Australia), an online systematic review software package. Using Covidence, 2 authors (either Janice Zhao, Maya Abdallah, Chandrika Sanapala, or Erin Watson) independently reviewed all titles and abstracts for eligibility (Fig. 1). Disagreements about eligibility were resolved by a third reviewer. If the same study was published as a clinical trial protocol, an abstract, and a full text, we extracted information from the full text only.

After the initial screening, 2 reviewers (Janice Zhao and Maya Abdallah) independently reviewed the remaining studies deemed eligible. At this stage, if a study had full text or a study protocol available, we reviewed the study in its entirety. To determine whether there was a full text associated with an abstract or study protocol, we searched PubMed and Google using the study titles. Disagreements were resolved by discussion. The references of selected full texts, abstracts, and study protocols were reviewed for additional studies.

### Data Extraction and Analysis

We created a data abstraction form to extract data from each study. Data extracted included first author, article type (ie, abstract, full text, and clinical trial protocol), study design, study population and sample size, decision aid format and content, adherence to International Patient Decision Aids Standards (IPDAS) criteria, decision type (eg, treatment choice, participation in clinical trials), study objective, and study outcomes. The data were extracted from the included studies by 2 reviewers independently, and disagreements were resolved by discussion. Data were summarized using descriptive statistics. We did not perform meta-analysis given the heterogeneity among the studies.

### Quality Assessment

We used the National Institutes of Health National Heart, Lung, and Blood Institute (NHLBI) study quality assessment tools to evaluate the quality of the articles^20^. Only studies with full text available were assessed using the NHLBI tool. Depending on the type of study, we used either the Quality Assessment of Controlled Intervention Studies tool or the Quality Assessment Tool for Before–After Studies tool. These tools evaluate the internal validity of a study and involve critical appraisal including the risk of potential for allocation bias, selection bias, information bias, measurement bias, and confounders. A high risk of bias translates to a rating of poor quality; low risk of bias translates to a rating of good quality. Studies were rated good, fair, or poor quality. Two reviewers (Janice Zhao and Maya Abdallah) independently completed assessments for each full-text article (Supplementary Table S2). Disagreements were resolved by discussion. To assess inter-rater reliability, we used the kappa statistic (0-0.2 slight agreement, 0.21-0.40 fair agreement, 0.41-0.60 moderate agreement, 0.61-0.90 substantial agreement, and 0.81-1 almost perfect).

## Results

### Study Characteristics

Using our search strategy, we initially identified 7693 studies (Fig. 1). Of these, we removed 2412 duplicates, resulting in 5281 unique titles and abstracts which we then screened using Covidence. After the initial screening, we identified and reviewed 27 studies for inclusion/eligibility. Ultimately, we included 15 studies for analysis in this systematic review (Tables 1 and 2). One study was only available as an abstract at the time of our initial search; however, the full text was published during our review process and we therefore included the full text in our review^21^. We also found one full text associated with an abstract^22^ and 2 full texts^23,24^ associated with clinical trial protocols that were not initially identified by our search strategy. For these studies, the full text was reviewed as opposed to the abstract or clinical trial protocol.

**Table 1.** Studies examining decision aids in hematologic malignancies.

Article	Publication type	Design and main objective	Population and sample size (if known)	Decision aid format	Decision aid content	IPDAS criteria	Decision type	Quality
Ahmad (2016)^28^	Abstract	Developmental	CML, CLL, high grade lymphoma, low grade lymphoma, multiple myeloma, or Hodgkin’s lymphoma	Video	Facts about each disease and management	No	Management	n/a
Dharmarajan (2019)^25^	Full	Single arm pilot (development and preliminary efficacy)	Lymphoma (*n* = 2) and solid tumors (*n* = 38)	Video	Process of radiation simulation, what to expect at time of treatment, side effects, and purpose of palliative care	Yes	Treatment, supportive care, palliative care	Good
Fadem (2018)^29^	Abstract	Developmental (survey for feedback on decision aid)	AML (*n* = 19), caregivers (*n* = 13)	Video-based decision support system, video clips	Patients shared their experiences and reflections based on the treatment they received and outcomes experienced	No	Treatment	n/a
Faiman (2019)^30^	Abstract	Developmental	Multiple myeloma, nurses	12 question PDA	Basic disease overview, series of questions	Yes	Treatment	n/a
Hildenbrand (2020)^21^	Full	Single arm pilot (feasibility and preliminary efficacy)	AML (*n* = 20)	Electronic with 10 animated videos	Disease overview, general treatment paths	Yes	Treatment, supportive care	Good
LeBlanc (2015)^31^	Abstract	Developmental (needs assessment)	Mantle cell lymphoma, healthcare providers	Online aid with graphics and audio	Risks and benefits of treatment options	Yes	Treatment	n/a
LeBlanc (2016)^32^	Abstract	Developmental (determine decision drivers)	CLL, mantle cell lymphoma, healthcare providers	Online aid with graphics and audio	Risks and benefits of treatment options	Yes	Treatment	n/a
Meropol (2016)^23^	Full	RCT (efficacy)	Various cancers (*n* = 1255) (hematologic malignancies presumed to fall under the “other” category based on study protocol)	Web-based, tailored, interactive computer program	Videos designed to address knowledge and attitudinal barriers of patients	Yes	Clinical trials as a treatment option	Fair
Miller (2009)^33^	Abstract	Single arm pilot (preliminary efficacy)	Multiple myeloma (*n* = 14), leukemia (*n* = 11), non-Hodgkin lymphoma (*n* = 10), or Hodgkin lymphoma (n = 2)	Written questionnaires	Decision model to prompt patients to list questions about their diagnosis and treatment	No	Treatment	n/a
Rocque (2018)^22^	Full	Single arm pilot (preliminary efficacy)	CLL (*n* = 44), lay navigators (n = 33), HCP (*n* = 54)	Combination of print, video, in-person lectures, online case-based education	CLL biology, treatment options, side effects, questions to ask the oncologist	No	Treatment	Good
Sebban (1995)^26^	Full	Developmental (design and validation)	Healthy personnel (*n* = 42)	Bedside decision instrument - decision board	Scenarios that describe treatment options for patients with CML; included information on procedures, morbidity, mortality	No	Treatment	Fair
Stevenson (2020)^24^	Full	RCT (effectiveness)	AML, ALL, Burkitt lymphoma or lymphoblastic lymphoma (*n* = 60), support persons (*n* = 15)	Web-based information, nurse delivered telephone support	Cancer and its causes, impact of cancer and treatment, available support services, links to relevant websites, discussion forum	No	Treatment and palliative approaches	Fair
Stienen (2015)^27^	Full	Single arm pilot (preliminary efficacy)	Non-Hodgkin lymphoma (*n* = 6), laymen (*n* = 6)	E-tool	Personal section for patients’ own experiences and informative section (general information, diagnostic exams, diagnosis, therapy, aftercare, and waiting times)	No	Management of care, including treatment (surveillance, radiotherapy, chemotherapy, immune therapy, stem cell transplantation, and clinical trials)	Poor
Woodard (2017)^35^	Protocol	RCT (efficacy)	Lymphoma, plasma cell myeloma, and other cancers (including solid cancers)	Website	–	No	Fertility preservation decisions	n/a
Wujcik (2020)^34^	Abstract	Single arm pilot (feasibility)	Non-Hodgkin lymphoma (*n* = 45)	Electronic tool	Tool included questions about needs, decision making preferences, values, and goals of care	No	Treatment decisions	n/a

Abbreviations: AML, acute myeloid leukemia; CLL, chronic lymphocytic leukemia; CML, chronic myeloid leukemia; HCP, healthcare providers; PRE-ACT, Preparatory Education about Clinical Trials; RT, radiation therapy.

**Table 2.** Outcomes of studies examining decision aids in hematologic malignancies.

Study phase	Article	Feasibility/acceptability	Preliminary efficacy	Quality
Developmental	Ahmad (2016)^28^	• 94% of patients showed interest in a short video on their disease; those who were not interested were >75 years of age and were not computer-users • 82% of patients who were interested in audio-visual information were also interested in other experiences of other patients		n/a
Developmental	Fadem (2018)^29^ (survey for feedback on decision aid)	• Determined 3 types of information needs: type, presentation, and access • Video clips were personalized to user based on their profiles, diagnosis, and treatment options		n/a
Developmental	Faiman (2019)^30^	• Common themes noted were the emphasis on the balance between quality and quantity of life as well as willingness to accept side effects rather than symptoms • 12 question PDA was developed that included disease overview and a series of questions		n/a
Developmental	LeBlanc (2015)^31^ (needs assessment)	• Patients identified efficacy, adverse effects, cost, and impact on daily life as major decision determinants • Patients prefer online format of PDA • HCPs felt patients chose treatment based on cost, efficacy, and impact on daily life		n/a
Developmental	LeBlanc (2016)^32^ (determine decision drivers)	• Patients identified efficacy, quality of life, and dosing schedule as major decision drivers • HCPs felt decision drivers for patients were quality of life, treatment toxicity, and efficacy •Patients felt the PDAs were helpful and informative		n/a
Developmental	Sebban (1995)^26^ (effectiveness)	• Instrument was feasible and acceptable for use in healthy individuals • Preferences elicited by the bedside instrument appeared to be reliable and valid		Fair
Single-arm pilot	Dharmarajan (2019)^25^ (development and preliminary efficacy)	• Patients felt comfortable watching the video (70%) and would highly recommend it to others (75%)	• Decision uncertainty was reduced • Palliative RT knowledge improved • Palliative RT readiness increased • Readiness for palliative care consultation was unchanged	Good
Single-arm pilot	Hildenbrand (2020)^21^ (feasibility and preliminary efficacy)	• Feasibility was met	• Knowledge scores improved • Decisional conflict scores were reduced	Good
Single-arm pilot	Miller (2009)^33^ (preliminary efficacy)		•Trend for decrease in distress, anxiety •Increase in patients’ confidence in knowing which questions to ask their doctor	n/a
Single-arm pilot	Rocque (2018)^22^ (preliminary efficacy)		• Increased Patient Activation Measure score after education • Increase in percentage of patients desiring and perceiving shared decision making • Increase in percentage of patients understanding that CLL is incurable • Increased awareness of signs of disease progression • Increased patient satisfaction with their oncologist’s explanation of therapy	Good
Single-arm pilot	Stienen (2015)^27^ (preliminary efficacy)		• At least 75% satisfaction on each feedback item (including use of e-tool, format of e-tool, and understandable information)	Poor
Single-arm pilot	Wujcik (2020)^34^ (feasibility)	• 47% prefer their doctor share responsibility with them when making treatment decisions • 67% said they wanted all the facts, but not prognosis • 24% concordance between patient and provider perception of how treatment decisions were made • Collecting patient preferences, values, and care goals prior to clinic visits using technology is feasible		n/a
RCT	Meropol (2016)^23^ (efficacy)		• Patients allocated to PRE-ACT showed greater increase in knowledge and greater decrease in attitudinal barriers • PRE-ACT was associated with greater patient satisfaction • Trend favoring the PRE-ACT group with regards to increased preparedness to consider clinical trials	Fair
RCT	Stevenson (2020)^24^ (effectiveness)	• Telephone support not utilized	• No statistically significant difference in unmet information needs, depression, or anxiety	Fair
RCT	Woodard (2017)^35^ (efficacy)			n/a

Of the 15 studies we included, 7 studies had full text available^21-27^, 7 studies were abstracts only^28-34^, and 1 was a study protocol^35^. Study designs included 6 PDA development studies^26,28-32^, 6 pilot studies^21,22,25,27,33,34^, and 3 randomized trials^23,24,35^. Six of the 15 studies implemented the IPDAS criteria when designing or evaluating their PDA^21,23,25,30-32^.

### Characteristics of PDAs and Participant

The hematologic malignancies represented in the studies included Non-Hodgkin lymphoma^22,24,27,28,31-34^, multiple myeloma (*n* = 4)^28,30,33,35^, and AML (*n* = 3)^21,24,29^ (Table 3). Among studies for which sample sizes of patients with hematologic malignancies were reported, the range was from 2 to 60. The PDAs in these studies addressed shared decision-making for treatment options such as chemotherapy^21,22,24,26,27,29^, hematopoietic stem cell transplantation^26,27,29^, and radiation therapy^25,27^. Other decisions addressed included supportive care decision making, including palliative care consultation^25^ and participation in clinical trials^23,24,27,33^. One study included a PDA designed for patients, but it was initially tested among healthy individuals (hospital staff and volunteers recruited from a blood donor clinic)^26^ (Table 2).

**Table 3.** Characteristics of studies.

Variables		Number of studies (*n* = 15)^a^
Publication type	Full text	7
	Abstract	7
	Protocol	1
Hematologic malignancies	Acute myeloid leukemia	3
	Myeloma	4
	Chronic lymphocytic leukemia	3
	Non-Hodgkin lymphoma	8^b^
	More than 1 type of hematologic malignancies and solid tumors	7
	Patients	14
Participants included	Caregivers	2
	Healthcare professionals	4
	Developmental	6
Study design	Single arm pilot	6
	Randomized controlled trial	3
	Electronic with web content, video, and/or audio	11
Patient decision aid format	Questionnaires	2
	Bedside instruments	1
	Combination of various formats	1
Decision type	Treatment options	13
	Clinical trial	4
	Supportive care	4
	Fertility option	1
	Knowledge	4
Outcomes assessed	Satisfaction	2
	Decisional conflict	3
	Anxiety/distress	3
	Attitudinal barriers	1

Note that numbers may add up to greater than 15 as studies may include multiple hematologic malignancies or may assess multiple outcomes.

Of the 8 studies that included patients with non-Hodgkin lymphoma, 1 included patients with CLL only, 1 included patients with mantle cell lymphoma only, and 6 included a combination of various non-Hodgkin lymphomas.

PDA formats included electronic with web content, videos, and/or audio (*n* = 11)^21,23-25,27-29,31,32,34,35^, questionnaires (*n* = 2)^30,33^, bedside instruments (*n* = 1)^26^, and a combination of various formats (*n* = 1)^22^. Additional characteristics of the individual studies are included in Table 2. Five of the 6 pilot studies had a high completion rate with at least 92% of participants who initially participated in a study completing it^21,22,25,27,34^. One pilot study (conducted by Miller et al., available in abstract form only) had lower completion: of the 37 patients who participated in the program, 14 patients had completed data^33^.

Average participant age in the studies ranged from 36.0 to 62.4 years. There were 1501 participants with gender reported: 643 were males (42.8%) and 858 were females (57.2%). The most commonly represented race was White, with 1159 participants (85.9%) identifying as White. Other races represented included Black (*n* = 20, 1.5%), Asian (*n* = 1, 0.1%), Alaskan Indiana/Alaskan Native (*n* = 1, 0.1%), and self-reported “non-white” (*n* = 165, 12.2%).

### Randomized Trials

Full texts for 2 of the 3 randomized control trials were available^23,24^. In the first study conducted by Meropol et al., 1255 patients with cancer were randomly assigned to use the Preparatory Education About Clinical Trials (PRE-ACT) or control groups^23^. Participants in the PRE-ACT group participated in a program that included an assessment of barriers to clinical trials, a values assessment, and access to a tailored library of videos intended to address both knowledge and attitudinal barriers of patients. Participants in the control group received a text that included the National Cancer Institute website that contains information on clinical trials. The study found that the PRE-ACT intervention was more effective than the control text in increasing knowledge and reducing attitudinal barriers (*P* < .001).

In the second study, Stevenson et al. conducted a randomized control trial that included 60 patients with newly diagnosed AML, acute lymphoblastic leukemia, Burkitt lymphoma, or lymphoblastic lymphoma as well as 15 support persons^24^. Patients and support persons were randomized to the intervention or control groups. Participants in the intervention group received access to a tailored, self-guided Web-based informational tool and they had access to an oncology nurse whom they could ask questions. Participants in the control group received usual care with generic texts providing psychosocial support information. The study did not find a statistically significant difference in unmet information needs, depression, or anxiety between arms. In addition, the telephone support service offered to the intervention arm was not utilized.

### Quality Assessment

Of the 7 studies with full texts available, the quality assessment determined that 3 were fair, 3 were good, and 1 was of poor quality. Both randomized control trials were rated as fair quality (Supplementary Table S2). Criteria that contributed to lower quality included lack of allocation concealment, lack of blinding of participants, lack of description regarding blinding in outcome assessment, drop out >20% from baseline to end of study, and lack of description of intervention adherence.

For studies with pre–post design, 1 was rated as fair, 3 were good, and 1 was of poor quality. Notably, most or all studies did not report whether the sample size in their study was large enough to provide confidence in their findings, did not provide description regarding blinding in outcome assessment, and generally only measured outcomes at one time point.

Kappa was 0.86 (CI, 0.77-0.96), indicating substantial agreement between raters.

### Outcomes

Important factors in treatment and supportive care decision making as identified by patients and caregivers in PDA developmental studies were efficacy, adverse effects, cost, and quality of life^31,32^. Outcomes assessed in studies included knowledge, satisfaction, decisional conflict, anxiety/distress, attitudinal barriers, and information needs (Table 2).

PDAs were associated with increased knowledge among participants in 4 studies. Patients showed increased knowledge about their disease, treatment options, and clinical trials^21-23,25^. One study aimed to assess patients’ knowledge of palliative radiation therapy and interest in palliative care consultation after watching a video decision aid. Using a 6 question survey (higher scores indicating higher knowledge levels), they found that patients’ knowledge scores increased from 60.4 (range 16.67-100) to 88.3 (range 33.33-100)^25^. Similarly, in a study on the use of an electronic decision aid in patients with AML, patient knowledge scores improved from an average of 11.8 out of 18 correct items to 15.1 out of 18 correct items with the use of the PDA^21^. In a study conducted by Rocque et al., the use of a PDA increased the percentage of patients understanding that chronic lymphocytic leukemia (CLL) is incurable (from 80% to 90%). In addition, the percentage of patients who reported an awareness of signs of disease progression increased from 64% to 76%^22^.

PDAs were found to be associated with increased patient satisfaction in 2 of the studies^22,23^. In one study, the use of an education program in patients with CLL was associated with a higher percentage of patients who were satisfied with their oncologist’s explanation of their treatment. This was assessed based on patients’ level of agreement with the statement “*I am satisfied with my doctor’s explanation of why I am getting this therapy instead of other available therapies*”^22^. Meropol et al. found that participants in the intervention arm reported greater levels of satisfaction with both the amount of information received and the presentation of the information^23^.

Additionally, PDAs were associated with decreased decisional conflict (*n* = 2)^21,25^ and attitudinal barriers to clinical trial participation (*n* = 1)^23^. Hildenbrand et al. found that with the use of a PDA, decisional conflict scores were reduced with a mean difference of −6.5 among patients with AML^21^.

The findings regarding the impact of PDAs on hematologic malignancies were not consistent across studies. Stevenson et al. did not find that their web-based tool resulted in a statistically significant reduction in unmet information needs, depression, or anxiety among participants. The authors noted that this may be, at least in part, due to an inadequate sample size. Additionally, the intervention included telephone support that was not utilized by participants in the study^24^. Although Hildenbrand et al. found that their PDA improved decisional conflict, there was not a significant improvement in anxiety scores of participants with AML after the use of the PDA^21^.

None of the studies specifically addressed the impact of PDAs on hematologic malignancies in older adults. One study found those aged over 75 and non-computer users reported less interest in the use of a short video as a PDA^28^.

## Discussion

Our systematic review identified 15 studies examining the use of PDAs for adult patients with hematologic malignancies and their caregivers. Of the 15 studies, 3 were randomized control trials and non-Hodgkin lymphoma was the most commonly investigated hematologic malignancy. Therefore, while current literature examining the use of PDAs for adults with hematologic malignancies is limited, the positive impact of PDAs on shared decision making and patient outcomes warrants additional research in this field.

PDAs have been shown to support shared decision making^5,6^. As cancer treatments advance, preference-sensitive and shared decision-making become increasingly relevant, especially in clinical scenarios where decision making is nuanced (eg, multiple acceptable treatment options)^36^. In a study of patients with non-Hodgkin lymphoma evaluating a patient preferences shared decision-making tool, 47% of participating patients preferred a shared decision-making approach with their physicians. Similarly, among patients with hematologic malignancies, the majority of patients preferred shared decision-making; only 3% preferred to make decisions without their doctor’s input and 6% preferred their doctor to make decisions without their input^37^.

Shared decision-making has been shown to improve patient satisfaction and promote patient adherence to treatment^4^. Shared decision-making is achieved when patients are fully informed of their treatment options and the risks/benefits of their options^38^. We found that PDAs increase patient knowledge, which helps them be more informed of the disease and its treatments options, thereby improving shared decision-making. Future studies are needed to evaluate the direct impact of decision aids on shared decision-making, using validated tools such as the shared decision-making (SDM-Q 9) questionnaire and OPTIONS scale^39,40^.

PDAs are available in a variety of formats including print and electronic. Accessibility should be considered when designing a PDA. While an online PDA allows patients to share information from the PDA with caregivers and friends more easily, some individuals may not have access to a computer/internet or may not be comfortable with using a computer to access the PDA^28^. Additional considerations should be made for patients with sensory deficits, such as visual or hearing impairments. The most common PDAs in the studies we examined in this review were online tools, some of which included interactive content and/or videos. Thus, it may be most helpful to consider offering alternative formats of PDAs to ensure that the PDAs are accessible to all patients and caregivers.

Systematic reviews evaluating PDAs in the treatment of solid tumors have shown mixed results^9,11^. For example, in a systematic review of the role of PDAs in early breast cancer treatment decision making, 33 studies were identified and demonstrated that PDAs helped improve knowledge and decrease decisional conflict^9^. Another systematic review examining the role of PDAs in treatment decision making among patients with colorectal cancer found that the number of studies and evidence was limited. The study identified 3 articles and 2 of these articles were rated to be of low quality^11^. Our findings are consistent with those of the latter systematic review demonstrating that PDAs are underutilized in hematologic malignancies, and data evaluating their use and benefits are limited.

The effect of PDAs on clinical workflow and amount of clinical involvement was generally not described in the studies. However, it should be noted that multiple PDAs in the studies involved an online tool that patients could access from home at their convenience, which is unlikely to disrupt the clinical workflow. Nonetheless, the implementation of PDAs in clinical work, including the perspectives of healthcare professionals, should be further studied.

Of note, there also was limited older adult participation in the studies included in this systematic review. For many hematologic malignancies, median age at diagnosis is at least 65 years^41^. However, of the studies for which mean or median participant age was reported, only 2 studies had an average participant age of 65 or greater^22,34^. Thus, the results of the studies evaluating PDAs may not be generalizable to patients seen in clinical practice. For instance, as noted by Ahman et al., patients aged over 75 and not computer users expressed less interest in a video format for a PDA^28^. Further studies evaluating and incorporating PDAs among older adult patients are warranted.

In addition to age, there was a limited representation of minority individuals in the studies (86% were white). Hematologic malignancies affect individuals of all races, and in some malignancies such as multiple myeloma, Black individuals are disproportionately affected compared to their white counterparts^42^. Racial disparities exist that contribute to decreased adherence to medical care as well as lower recruitment to clinical trials among minority patients. A systematic review examining the use of decision aids among minority patients found that culturally tailored decision aids were more likely to lead to changes in clinical decision^43^. Therefore, PDAs can help address health disparities but their development and testing should culturally tailored to minority patients.

Quality assessment of studies revealed challenges with allocation concealment, blinding of participants and in outcome assessment, sample size justification, drop out, and time points of assessments. Unlike therapeutic trials, it is inherently difficult for blinding of patients in randomized trials of PDAs. However, blinding of study staff responsible for randomization, data collection, and analysis should be strongly considered. Sample size justification should be included in all studies, including those of qualitative and single-arm studies. Reasons for drop out should also be collected and reported, so that characteristics of those who do and do not drop out can be compared to inform future adaptation of the study procedures and intervention. Finally, decision making is a continuing process and therefore assessments should be collected at multiple time points for analyses.

Our systematic review was limited by the number of studies examining PDAs for adult patients with hematologic malignancies and/or their caregivers. However, our findings also highlighted the scarcity of studies in this topic. Although our search strategy to identify studies was developed with the assistance of a medical librarian, it is possible that some studies may have been inadvertently omitted during our search. We searched studies by diagnosis and it is possible that studies on decision aids for a specific type of treatment [eg, stem cell transplantation (ie, stem cell transplantation, Chimeric antigen receptor T-cell therapy)] were missed. We were unable to perform meta-analyses given the heterogeneity in the study populations and outcome measures.

## Conclusion

Research on PDAs for adult patients with hematologic malignancies and their caregivers is limited. Among the studies available, PDAs appear to support patients in shared decision-making. Based on the findings of this systematic review, further research efforts are warranted, ideally through randomized control trials, to better evaluate the benefits and strategies of implementing PDAs in clinical care of adults with hematologic malignancies. This is especially important in preference-sensitive scenarios which are increasingly relevant in older adults in whom multiple patient– and disease–specific factors must be considered. Important areas where PDAs can support treatment decision making include intensive vs. non-intensive chemotherapy in AML, full-dose versus reduced dosed chemotherapy in diffuse large B-cell lymphoma, allogeneic stem cell transplantation, and chimeric antigen receptor T-cell therapy. Given the rapidly evolving treatment paradigms in many hematologic malignancies, it is important that these PDAs have the ability to incorporate up-to-date information, that they can be personalized to individuals based on patient-specific factors such as underlying health status and preferences and can be easily incorporated into the clinical workflow.

## Supplementary Material

oyac231_suppl_Supplementary_Material

## Data Availability

No new data were generated or analyzed in support of this research.

## Prior Presentation

The study was selected for poster presentation at the 2021 American Geriatrics Society Annual Meeting.
