# Supplementary material for: A Systematic Review of Decision Aids in Hematologic Malignancies: What Are Currently Available and What Are We Missing?
Source: Oncologist. 2022 Nov 7;28(2):105–15. doi: 10.1093/oncolo/oyac231 (PMC9907042; doi:10.1093/oncolo/oyac231)
Supplement: oyac231_suppl_Supplementary_Material [file oyac231_suppl_supplementary_material.docx]

**Supplemental Table 1: Search strategies**

| **Database** | **Search Strategy** | **Number of Results** |
| --- | --- | --- |
| **PubMed** | (**"Decision Support Techniques"[Mesh] OR "Decision Support Systems, Clinical"[Mesh] OR "Decision Making"[Mesh] OR "Decision Making, Computer-Assisted"[Mesh]** OR "Decision Support"[tiab] OR "Decision Supports"[tiab] OR "Decision Model"[tiab] OR "Decision Models"[tiab] OR "Decision Modeling"[tiab] OR "Decision Aid"[tiab] OR "Decision Aids"[tiab] OR "Decision Analysis"[tiab] OR "Decision Analyses"[tiab] OR "Decision Making"[tiab] OR "CDS"[tiab]) **AND** (**"Hematologic Neoplasms"[Mesh] OR "Neoplasms, Plasma Cell"[Mesh] OR "Leukemia"[Mesh] OR "Lymphoma"[Mesh]** OR "Hematologic Neoplasm"[tiab] OR "Hematologic Neoplasms"[tiab] OR "Hematologic Malignancy"[tiab] OR "Hematologic Malignancies"[tiab] OR "Hematologic Cancer"[tiab] OR "Hematologic Cancers"[tiab] OR "Hematological Neoplasm"[tiab] OR "Hematological Neoplasms"[tiab] OR "Hematological Malignancy"[tiab] OR "Hematological Malignancies"[tiab] OR "Hematological Cancer"[tiab] OR "Hematological Cancers"[tiab] OR "Hematopoietic Neoplasm"[tiab] OR "Hematopoietic Neoplasms"[tiab] OR "Hematopoietic Malignancy"[tiab] OR "Hematopoietic Malignancies"[tiab] OR "Hematopoietic Cancer"[tiab] OR "Hematopoietic Cancers"[tiab] OR "Bone Marrow Neoplasm"[tiab] OR "Bone Marrow Neoplasms"[tiab] OR "Bone Marrow Malignancy"[tiab] OR "Bone Marrow Malignancies"[tiab] OR "Bone Marrow Cancer"[tiab] OR "Bone Marrow Cancers"[tiab] OR "Plasma Cell Neoplasm"[tiab] OR "Plasma Cell Neoplasms"[tiab] OR "Plasma Cell Malignancy"[tiab] OR "Plasma Cell Malignancies"[tiab] OR "Plasma Cell Cancer"[tiab] OR "Plasma Cell Cancers"[tiab] OR "Myeloma"[tiab] OR "Myelomas"[tiab] OR "Myelomatosis"[tiab] OR "Myelomatoses"[tiab] OR "Leukemia"[tiab] OR "Leukemias"[tiab] OR "Leucocythaemia"[tiab] OR "Leucocythaemias"[tiab] OR "Leucocythemia"[tiab] OR "Leucocythemias"[tiab] OR "Lymphoma"[tiab] OR "Lymphomas"[tiab] OR "Germinoblastic Sarcoma"[tiab] OR "Germinoblastic Sarcomas"[tiab] OR "Reticulolymphosarcoma"[tiab] OR "Reticulolymphosarcomas"[tiab] OR "Germinoblastoma"[tiab] OR "Germinoblastomas"[tiab]) **AND** ("Patient"[tiab] OR "Patients"[tiab] OR "Client"[tiab] OR "Clients"[tiab] OR "Inpatient"[tiab] OR "Inpatients"[tiab] OR "Outpatient"[tiab] OR "Outpatients"[tiab] OR "Caregiver"[tiab] OR "Caregivers"[tiab] OR "Carer"[tiab] OR "Carers"[tiab] OR "Care Giver"[tiab] OR "Care Givers"[tiab] OR "Spouse"[tiab] OR "Spouses"[tiab] OR "Parent"[tiab] OR "Parents"[tiab]) **NOT** (("Adult"[Mesh]) NOT ("Adult"[Mesh] AND ("Adolescent"[Mesh] OR "Child"[Mesh] OR "Infant"[Mesh]))) | 1,696 |

| **Database** | **Search Strategy** | **Number of Results** |
| --- | --- | --- |
| **Embase** | (**'decision support system'/exp OR 'patient decision making'/exp OR 'patient decision aid'/exp** OR 'Decision Support':ti,ab OR 'Decision Supports':ti,ab OR 'Decision Model':ti,ab OR 'Decision Models':ti,ab OR 'Decision Modeling':ti,ab OR 'Decision Aid':ti,ab OR 'Decision Aids':ti,ab OR 'Decision Analysis':ti,ab OR 'Decision Analyses':ti,ab OR 'Decision Making':ti,ab OR 'CDS':ti,ab) **AND** (**'hematologic malignancy'/exp** OR 'Hematologic Neoplasm':ti,ab OR 'Hematologic Neoplasms':ti,ab OR 'Hematologic Malignancy':ti,ab OR 'Hematologic Malignancies':ti,ab OR 'Hematologic Cancer':ti,ab OR 'Hematologic Cancers':ti,ab OR 'Hematological Neoplasm':ti,ab OR 'Hematological Neoplasms':ti,ab OR 'Hematological Malignancy':ti,ab OR 'Hematological Malignancies':ti,ab OR 'Hematological Cancer':ti,ab OR 'Hematological Cancers':ti,ab OR 'Hematopoietic Neoplasm':ti,ab OR 'Hematopoietic Neoplasms':ti,ab OR 'Hematopoietic Malignancy':ti,ab OR 'Hematopoietic Malignancies':ti,ab OR 'Hematopoietic Cancer':ti,ab OR 'Hematopoietic Cancers':ti,ab OR 'Bone Marrow Neoplasm':ti,ab OR 'Bone Marrow Neoplasms':ti,ab OR 'Bone Marrow Malignancy':ti,ab OR 'Bone Marrow Malignancies':ti,ab OR 'Bone Marrow Cancer':ti,ab OR 'Bone Marrow Cancers':ti,ab OR 'Plasma Cell Neoplasm':ti,ab OR 'Plasma Cell Neoplasms':ti,ab OR 'Plasma Cell Malignancy':ti,ab OR 'Plasma Cell Malignancies':ti,ab OR 'Plasma Cell Cancer':ti,ab OR 'Plasma Cell Cancers':ti,ab OR 'Myeloma':ti,ab OR 'Myelomas':ti,ab OR 'Myelomatosis':ti,ab OR 'Myelomatoses':ti,ab OR 'Leukemia':ti,ab OR 'Leukemias':ti,ab OR 'Leucocythaemia':ti,ab OR 'Leucocythaemias':ti,ab OR 'Leucocythemia':ti,ab OR 'Leucocythemias':ti,ab OR 'Lymphoma':ti,ab OR 'Lymphomas':ti,ab OR 'Germinoblastic Sarcoma':ti,ab OR 'Germinoblastic Sarcomas':ti,ab OR 'Reticulolymphosarcoma':ti,ab OR 'Reticulolymphosarcomas':ti,ab OR 'Germinoblastoma':ti,ab OR 'Germinoblastomas':ti,ab) **AND** ('Patient':ti,ab OR 'Patients':ti,ab OR 'Client':ti,ab OR 'Clients':ti,ab OR 'Inpatient':ti,ab OR 'Inpatients':ti,ab OR 'Outpatient':ti,ab OR 'Outpatients':ti,ab OR 'Caregiver':ti,ab OR 'Caregivers':ti,ab OR 'Carer':ti,ab OR 'Carers':ti,ab OR 'Care Giver':ti,ab OR 'Care Givers':ti,ab OR 'Spouse':ti,ab OR 'Spouses':ti,ab OR 'Parent':ti,ab OR 'Parents':ti,ab) **NOT** ('juvenile'/exp NOT ('juvenile'/exp AND 'adult'/exp)) | 2,879 |

| **Database** | **Search Strategy** | **Number of Results** |
| --- | --- | --- |
| **Web of Science** | ("Decision Support" OR "Decision Supports" OR "Decision Model" OR "Decision Models" OR "Decision Modeling" OR"Decision Aid" OR "Decision Aids" OR "Decision Analysis" OR "Decision Analyses" OR "Decision Making" OR "CDS") **AND** ("Hematologic Neoplasm" OR "Hematologic Neoplasms" OR "Hematologic Malignancy" OR "Hematologic Malignancies" OR "Hematologic Cancer" OR "Hematologic Cancers" OR "Hematological Neoplasm" OR "Hematological Neoplasms" OR "Hematological Malignancy" OR "Hematological Malignancies" OR "Hematological Cancer" OR "Hematological Cancers" OR "Hematopoietic Neoplasm" OR "Hematopoietic Neoplasms" OR "Hematopoietic Malignancy" OR "Hematopoietic Malignancies" OR "Hematopoietic Cancer" OR "Hematopoietic Cancers" OR "Bone Marrow Neoplasm" OR "Bone Marrow Neoplasms" OR "Bone Marrow Malignancy" OR "Bone Marrow Malignancies" OR "Bone Marrow Cancer" OR "Bone Marrow Cancers" OR "Plasma Cell Neoplasm" OR "Plasma Cell Neoplasms" OR "Plasma Cell Malignancy" OR "Plasma Cell Malignancies" OR "Plasma Cell Cancer" OR "Plasma Cell Cancers" OR "Myeloma" OR "Myelomas" OR "Myelomatosis" OR "Myelomatoses" OR "Leukemia" OR "Leukemias" OR "Leucocythaemia" OR "Leucocythaemias" OR "Leucocythemia" OR "Leucocythemias" OR "Lymphoma" OR "Lymphomas" OR "Germinoblastic Sarcoma" OR "Germinoblastic Sarcomas" OR "Reticulolymphosarcoma" OR "Reticulolymphosarcomas" OR "Germinoblastoma" OR "Germinoblastomas") **AND** ("Patient" OR "Patients" OR "Client" OR "Clients" OR "Inpatient" OR "Inpatients" OR "Outpatient" OR "Outpatients" OR "Caregiver" OR "Caregivers" OR "Carer" OR "Carers" OR "Care Giver" OR "Care Givers" OR "Spouse" OR "Spouses" OR "Parent" OR "Parents") | 2,888 |

| **Database** | **Search Strategy** | **Number of Results** |
| --- | --- | --- |
| **Cochrane Central Register of Controlled Trials** | ("Decision Support" OR "Decision Supports" OR "Decision Model" OR "Decision Models" OR "Decision Modeling" OR "Decision Aid" OR "Decision Aids" OR "Decision Analysis" OR "Decision Analyses" OR "Decision Making" OR "CDS") **AND** ("Hematologic Neoplasm" OR "Hematologic Neoplasms" OR "Hematologic Malignancy" OR "Hematologic Malignancies" OR "Hematologic Cancer" OR "Hematologic Cancers" OR "Hematological Neoplasm" OR "Hematological Neoplasms" OR "Hematological Malignancy" OR "Hematological Malignancies" OR "Hematological Cancer" OR "Hematological Cancers" OR "Hematopoietic Neoplasm" OR "Hematopoietic Neoplasms" OR "Hematopoietic Malignancy" OR "Hematopoietic Malignancies" OR "Hematopoietic Cancer" OR "Hematopoietic Cancers" OR "Bone Marrow Neoplasm" OR "Bone Marrow Neoplasms" OR "Bone Marrow Malignancy" OR "Bone Marrow Malignancies" OR "Bone Marrow Cancer" OR "Bone Marrow Cancers" OR "Plasma Cell Neoplasm" OR "Plasma Cell Neoplasms" OR "Plasma Cell Malignancy" OR "Plasma Cell Malignancies" OR "Plasma Cell Cancer" OR "Plasma Cell Cancers" OR "Myeloma" OR "Myelomas" OR "Myelomatosis" OR "Myelomatoses" OR "Leukemia" OR "Leukemias" OR "Leucocythaemia" OR "Leucocythaemias" OR "Leucocythemia" OR "Leucocythemias" OR "Lymphoma" OR "Lymphomas" OR "Germinoblastic Sarcoma" OR "Germinoblastic Sarcomas" OR "Reticulolymphosarcoma" OR "Reticulolymphosarcomas" OR "Germinoblastoma" OR "Germinoblastomas") **AND** ("Patient" OR "Patients" OR "Client" OR "Clients" OR "Inpatient" OR "Inpatients" OR "Outpatient" OR "Outpatients" OR "Caregiver" OR "Caregivers" OR "Carer" OR "Carers" OR "Care Giver" OR "Care Givers" OR "Spouse" OR "Spouses" OR "Parent" OR "Parents") | 224 |
| **ClinicalTrials.gov** | ("Decision Support" OR "Decision Supports" OR "Decision Model" OR "Decision Models" OR "Decision Modeling" OR "Decision Aid" OR "Decision Aids" OR "Decision Analysis" OR "Decision Analyses" OR "Decision Making" OR "CDS") **AND** (((Hematolog* OR (Bone Marrow) OR (Plasma Cell*)) AND (Neoplas* OR Malignan* OR Cancer*)) OR Myeloma* OR Leukemia* OR Leucocythaemia* OR Leucocythemia* OR Lymphoma* OR (Germinoblastic Sarcoma*) OR Reticulolymphosarcoma* OR Germinoblastomas*) | 6 |

**Supplemental Table 2. Example of quality assessment ^14^; possible quality ratings of the studies were good, fair, or poor.**

| **Criteria** | **Meropol et al. 2016 ^17^** | **Stevenson et al. 2020 ^18^** |
| --- | --- | --- |
| 1. Was the study described as randomized, a randomized trial, a randomized clinical trial, or an RCT? | Yes | Yes |
| 2. Was the method of randomization adequate (i.e., use of randomly generated assignment)? | Yes | Yes |
| 3. Was the treatment allocation concealed (so that assignments could not be predicted)? | Yes | No |
| 4. Were study participants and providers blinded to treatment group assignment? | Not Reported | No |
| 5. Were the people assessing the outcomes blinded to the participants' group assignments? | Not Reported | Yes |
| 6. Were the groups similar at baseline on important characteristics that could affect outcomes (e.g., demographics, risk factors, co-morbid conditions)? | Yes | Yes |
| 7. Was the overall drop-out rate from the study at endpoint 20% or lower of the number allocated to treatment? | No | No |
| 8. Was the differential drop-out rate (between treatment groups) at endpoint 15 percentage points or lower? | Yes | Yes |
| 9. Was there high adherence to the intervention protocols for each treatment group? | Not Applicable | Not Reported |
| 10. Were other interventions avoided or similar in the groups (e.g., similar background treatments)? | Yes | Yes |
| 11. Were outcomes assessed using valid and reliable measures, implemented consistently across all study participants? | Not Reported | Yes |
| 12. Did the authors report that the sample size was sufficiently large to be able to detect a difference in the main outcome between groups with at least 80% power? | Yes | Yes |
| 13. Were outcomes reported or subgroups analyzed prespecified (i.e., identified before analyses were conducted)? | Yes | Yes |
| 14. Were all randomized participants analyzed in the group to which they were originally assigned, i.e., did they use an intention-to-treat analysis? | Yes | Yes |
|  |  |  |
| Number of Yes: | 9 | 10 |
| Quality Rating: | Fair | Fair |
